# DGNMDA: Dual Heterogeneous Graph Neural Network Encoder for miRNA-Disease Association Prediction

**DOI:** 10.3390/bioengineering11111132

**Published:** 2024-11-10

**Authors:** Daying Lu, Qi Zhang, Chunhou Zheng, Jian Li, Zhe Yin

**Affiliations:** 1 School of Cyber Science and Engineering, Qufu Normal University, Qufu 273165, China; zhangqi12252024@163.com (Q.Z.); zhengchung99@126.com (C.Z.); jianli0419@126.com (J.L.); dayinglu1@qfnu.edu.cn (Z.Y.); 2 Artificial Intelligence Academy, Anhui University, Hefei 230039, China

**Keywords:** miRNA-disease association prediction, graph convolutional transformer, feature interaction gating, gcan

## Abstract

In recent years, numerous studies have highlighted the pivotal importance of miRNAs in personalized healthcare, showcasing broad application prospects. miRNAs hold significant potential in disease diagnosis, prognosis assessment, and therapeutic target discovery, making them an integral part of precision medicine. They are expected to enable precise disease subtyping and risk prediction, thereby advancing the development of precision medicine. GNNs, a class of deep learning architectures tailored for graph data analysis, have greatly facilitated the advancement of miRNA-disease association prediction algorithms. However, current methods often fall short in leveraging network node information, particularly in utilizing global information while neglecting the importance of local information. Effectively harnessing both local and global information remains a pressing challenge. To tackle this challenge, we propose an innovative model named DGNMDA. Initially, we constructed various miRNA and disease similarity networks based on authoritative databases. Subsequently, we creatively design a dual heterogeneous graph neural network encoder capable of efficiently learning feature information between adjacent nodes and similarity information across the entire graph. Additionally, we develop a specialized fine-grained multi-layer feature interaction gating mechanism to integrate outputs from the neural network encoders to identify novel associations connecting miRNAs with diseases. We evaluate our model using 5-fold cross-validation and real-world disease case studies, based on the HMDD V3.2 dataset. Our method demonstrates superior performance compared to existing approaches in various tasks, confirming the effectiveness and potential of DGNMDA as a robust method for predicting miRNA-disease associations.

## 1. Introduction

miRNAs, small non-coding RNAs, are key regulators of post-transcriptional gene expression. Despite their short length of 19 to 24 nucleotides, miRNAs can simultaneously regulate the expression of numerous genes through their broad targeting ability, modulating the expression of a vast array of mRNAs, especially those genes participating in interrelated biological pathways, allowing them to precisely adjust gene expression and orchestrate intricate cellular processes [1]. In 2002, Gregory J. Hannon and his research team [2] first reported the aberrant expression of two human miRNAs, let-7 [3] and lin-4 [4], in human lung cancer cells [5], laying the foundation for further exploration of the link between miRNAs and cancer [6], as well as other diseases, and subsequently, research on miRNAs as disease biomarkers and potential therapeutic targets gradually deepened. Traditional methods for predicting miRNA-disease associations relied on statistical analysis, the use of known biomarkers, and biological experimental validation. However, these methods often faced challenges such as difficulties in data acquisition, limited prediction accuracy, and applicability to only specific types of diseases, making them difficult to generalize. With advancements in technology and the continuous development of bioinformatics, researchers have developed more comprehensive modern methods aimed at achieving in-depth analysis of disease pathogenesis and accurate prediction of miRNA-disease associations. Current approaches mainly fall into two classes: those based on network analysis and those employing machine learning techniques.

Methods relying on network analysis typically build heterogeneous miRNA-disease networks by assessing the commonalities between miRNAs and diseases, along with their association matrices. They utilize the similarity information within the network to uncover and infer novel miRNA-disease associations [7]. Xie et al. [8] integrated data from various biological perspectives to develop a comprehensive miRNA-disease network, enhancing the accuracy and reliability of predictions. Yu et al. [9] employed a combination of tensor decomposition and label propagation techniques. They used tensor decomposition to extract crucial biological information, simplifying the analysis of intricate interactions between miRNAs and diseases. Wang et al. [10] introduced an attention-based graph convolution approach to identify disease-related miRNAs. The precision of network-based methods heavily depends on calculating the similarity between miRNAs and diseases, with the quality and completeness of prior knowledge directly impacting prediction performance. When prior knowledge is limited or unreliable, the accuracy of prediction results may be affected.

Machine learning-based methods involve using trained machine learning models to predict unknown data and identify potential miRNA-disease links. To demonstrate this, Chen et al. [11] utilized existing miRNA-disease links data and improved prediction accuracy by incorporating neighborhood constraints into the matrix completion process. Zhou et al. [12] employed gradient boosting decision trees to extract features and then used logistic regression for feature scoring and classification, this enables the prediction of potential links connecting miRNAs to various diseases. Xuan et al. [13] developed dual convolutional neural networks to analyze miRNA and disease data, adeptly identifying the intricate relationships and patterns inherent in these datasets. In summary, machine learning-based association prediction models have demonstrated remarkable efficiency. These models not only significantly reduce time and financial costs but also rely heavily on the quality of the extracted features for the prediction results. Therefore, extracting high-quality, information-rich features from raw data is crucial for improving prediction performance. Future research should concentrate on developing advanced feature engineering techniques to further enhance the performance of these models in predicting miRNA-disease associations.

The rapid development of graph neural networks (GNNs) [14] and transformers [15,16,17,18] has led to various GNN models, such as Graph Convolutional Networks (GCNs) [19], Graph Attention Networks (GATs) [20], and Graph Autoencoders (GAEs) [21]. Tang et al. [22] introduced MMGCN, a deep learning approach that employs GCN encoders to derive miRNA and disease representations from various similarity perspectives. It further refines these features using multi-channel attention to fill in the missing entries in the miRNA-disease association matrix. HGANMDA [23], a hierarchical graph attention network, predicts RNA-disease associations by leveraging attention mechanisms at both node and semantic levels to capture the significance of adjacent nodes and various meta-paths. Zhou et al. [24] introduced a method based on a multi-molecule heterogeneous graph transformer, integrating biological entity relationships of eight major biomolecules to construct a comprehensive heterogeneous biological entity graph and using a heterogeneous transformer for miRNA prediction. Despite the remarkable performance of these models in prediction tasks, they often have limitations in leveraging network node information, particularly in emphasizing global information while overlooking the importance of local information. Efficiently considering both local and global information remains a pressing challenge. Moreover, existing models solely consider the relationship attributes between node pairs when leveraging heterogeneous network data, posing an additional challenge to address. Inspired by L. H. Torres et al. [25], we propose DGNMDA, an innovative dual heterogeneous graph neural network encoder designed to predict miRNA-disease associations.

**Figure 1:**
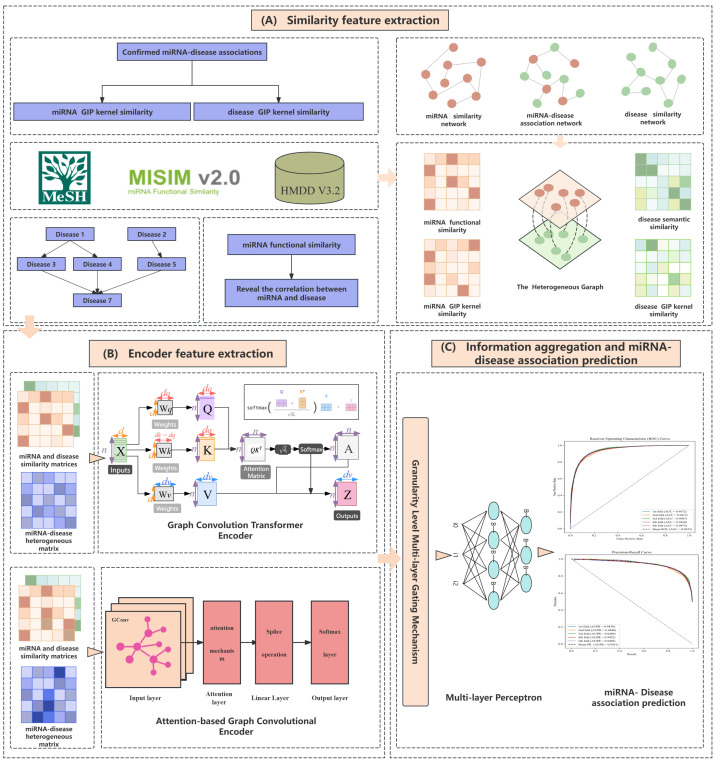
Overall architecture of DGNMDA.

Initially, we build miRNA and disease homogeneous networks, as well as a miRNA-disease heterogeneous similarity network, leveraging various similarities among miRNAs and diseases. These networks comprehensively characterize the complex relationships and interactions between miRNAs and diseases. Then, we innovatively design a dual heterogeneous graph neural network encoder, consisting of a Graph Convolutional Transformer and a Graph Convolutional Attention Network (GCAN) encoder. The Graph Convolutional Transformer encoder captures both local structural information and global dependencies of nodes by introducing graph convolutional layers and self-attention mechanisms. The GCAN encoder adaptively aggregates information from neighboring nodes through an attention mechanism, effectively learning the local structural information of nodes. Through the collaborative work of these two encoders, our approach enables us to thoroughly capture local and global characteristics, obtaining high-quality node-embedding encodings. To further improve the quality of feature representations and prediction performance, we design a fine-grained multi-layer gating mechanism. This mechanism adaptively fuses and refines feature representations from the Graph Convolutional Transformer encoder and GCAN encoder at different granularity levels through gating units. This layer-wise progressive feature fusion approach effectively combines the strengths of both encoders, generating more refined and discriminative feature representations. Finally, we input the fused feature embeddings into a multi-layer perceptron (MLP) to predict the association scores between miRNAs and diseases. The overall workflow of our method is shown in Figure 1.

In this paper, we propose DGNMDA, a dual heterogeneous graph neural network encoder specifically designed for predicting miRNA-disease associations. Our method makes contributions in the following aspects:

- Combining local structural information and global dependencies: We design a dual heterogeneous graph neural network encoder that integrates a Graph Convolutional Transformer and a Graph Convolutional Attention Network (GCAN). This architecture not only captures the global dependencies of nodes but also effectively learns local structural information, generating more comprehensive node embedding encodings.
- Adaptive fusion of multi-level features: We introduce a fine-grained feature interaction gating mechanism to gradually fuse and refine feature representations from the two encoders at different levels. This adaptive fusion mechanism allows the model to dynamically adjust feature combinations based on task requirements, improving the flexibility and prediction performance of the model.
- Improving prediction performance: Through experimental validation on the miRNA-disease association prediction task, our DGNMDA method demonstrates significant performance improvements on multiple benchmark datasets. Our results demonstrate the efficacy and advantages of our method, offering novel insights and resources for future studies in this domain.

## 2. Materials and Methods

### 2.1. Experimental Data

HMDD, a comprehensive database, offers a robust basis for investigating miRNA-human disease associations. This database integrates extensively validated miRNA-disease links, covering a wide range of laboratory research findings and clinical research discoveries. In this study, for an unbiased model comparison, we employ the HMDD v3.2 benchmark dataset, comprising 12,446 established links among 853 miRNAs and 591 diseases. We label these associations as positive instances. To tackle the uneven data distribution, we perform undersampling on the negative samples. Specifically, we equalize the dataset by randomly choosing an equivalent count of 0-labeled instances. This strategy ensures that the model is exposed to an equal proportion of positive and negative instances throughout training, consequently enhancing its resilience and generalizability. For a thorough assessment of our method’s efficacy, we carefully design data splitting and model evaluation strategies. First, we split the data into training and testing subsets in an 8:2 proportion to assess the model’s generalization ability. During the training phase, we utilize 5-fold CV to optimize the model’s hyperparameters and structural settings. By doing so, we can comprehensively evaluate the model’s performance on different data subsets and select the best-performing model configuration for the final independent testing, ensuring reliable prediction results in real-world applications.

### 2.2. Building miRNA-Disease Resemblance Graphs

To effectively represent miRNA and disease characteristics, we build separate homogeneous similarity networks and a combined heterogeneous network. When constructing the homogeneous similarity networks, we leverage the miRNA functional and GIP kernel similarity matrices to quantify miRNA similarities, while analogous techniques are used to build the disease homogeneous similarity network. For the heterogeneous affinity network, we incorporate validated miRNA-disease interaction data into a binary adjacency matrix of size M × N, with M and N denoting the miRNA and disease counts, respectively. This adjacency matrix enables us to obtain a miRNA-disease bipartite graph structure, in which miRNAs and diseases are represented as distinct node categories, and their known associations are represented as edges connecting the two node types. Finally, we derive the matrix form of the heterogeneous miRNA-Disease network, as shown below:

(1) $$G=\left[\begin{array}{cc} 0 & G_{md}\\ G_{dm}^{T} & 0 \end{array}\right]\in R^{\left(M+N)\times (M+N\right)}$$

where $G_{md}$ represents the miRNA-disease association sub-matrix, and $G_{dm}^{T}$ represents its transpose.

### 2.3. Graph Convolutional Attention Network (GCAN) Encoder

Graph Convolutional Networks (GCNs), a groundbreaking neural network architecture, are tailored for analyzing data with graph structures. GCNs ingeniously incorporate nodes topologically and attribute information by performing local convolutional operations on the graph to extract latent node representations.The core mechanism of GCNs utilizes the graph’s adjacency matrix to guide the propagation of information between nodes, accurately modeling the local neighborhood structure. In each network layer, GCNs adaptively aggregate and transform the features of neighboring nodes, a process that can be viewed as spectral filtering of graph signals. As the network depth increases, the model gradually expands its receptive field, capturing broader structural information. This hierarchical feature extraction approach enables GCNs to simultaneously encode both microscopic local structures and macroscopic global patterns, ultimately generating node embeddings rich in graph topological semantics. Inspired by previous work, we employ a Graph Convolutional Attention Network (GCAN) for encoding. Our GCAN model first utilizes a traditional GCN to perform initial encoding on the input similarity network, providing rich local structural information and better feature initialization for the subsequent attention mechanism. Then, we input the encoded features into the attention-based layer. This design allows the model to adaptively assign importance weights to different neighboring nodes, capturing long-range dependencies between nodes while preserving the local topological structure. The detailed procedure is outlined below:

First, we employ GCN to encode the node features. For node $v_{i}$, the following equation describes the feature update process:

(2) $$h_{v_{i}}^{\left(l\right)}=\sigma \left(\sum_{j\in N(i)\cup \{i\}}\frac{1}{\sqrt{\left|N(i)||N(j)\right|}}W^{\left(l\right)}h_{\nu_{j}}^{\left(l-1\right)}\right)$$

where $h{v_{i}}^{\left(l\right)}$ denotes the feature representation of node $v_{i}$ at the *l*-th layer, and N(i) represents its neighbor set, $W^{\left(l\right)}$ is the learnable weight matrix of the *l*-th layer, and σ is the ReLU activation function.

To distinguish the importance of different neighboring nodes, we introduce an attention mechanism. For node pair $\left(v_{i},v_{j}\right)$, we calculate the attention coefficient $e_{ij}$, normalizing the attention coefficients yields the ultimate attention weights $\alpha_{ij}$, and use the computed attention weights to perform weighted aggregation of the neighboring nodes’ features.

(3) $$e_{ij}=LeakyReLU\;\left(a^{T}\left[W_{h}h_{v_{i}}\|W_{h}h_{v_{j}}\right]\right)$$

(4) $$\alpha_{ij}=\frac{\exp\left(e_{ij}\right)}{\sum_{k\in N(i)}\exp\left(e_{ik}\right)}$$

(5) $$z_{v_{i}}=\sigma \left(\sum_{j\in N(i)}\alpha_{ij}W_{h}h_{v_{j}}\right)$$

where *a* is the learnable attention weight vector, $W_{h}$ is the shared feature transformation matrix, and || denotes vector concatenation. $z_{v_{i}}$ represents the new feature representation of node $v_{i}$ after attention-weighted aggregation.

To bolster the model’s expressiveness and robustness, we employ multi-head attention, computing separate attention heads concurrently and concatenating their outputs. Meanwhile, to mitigate the issue of vanishing gradients in deep networks while retaining useful information from each layer, we introduce an improved skip connection method. This method preserves the advantages of traditional skip connections while considering the issues of noise accumulation and feature importance, and has relatively low computational complexity, which is particularly important for processing large-scale graph data. Specifically, we fuse the outputs of multiple GCAN layers through the improved skip connections.

(6) $$h_{v_{i}}^{\mathrm{att}}{=\|}_{q=1}^{Q}\sigma \left(\sum_{j\in N(i)}\alpha_{ij}^{q}W_{h}^{q}h_{v_{j}}\right)$$

(7) $$H_{AG}=h_{v_{i}}^{\left(L\right)}+\sum_{l=1}^{L-1}\alpha_{l}h_{v_{i}}^{\left(l\right)}$$

where $\alpha_{ij}^{q}$ and $W_{h}^{q}$ denote the attention weights and feature transformation matrix of the *q*-th attention head, respectively, with *L* denoting the GCAN layer count $\alpha_{l}$ representing the *l*-th layer’s attention weight, which can be obtained through learning.

### 2.4. Graph Convolutional Transformer Encoder

Here, we adopt a Graph Convolutional Transformer for information extraction. Unlike traditional Transformers, the Graph Convolutional Transformer introduces graph convolutional layers to model the local structural information of nodes while leveraging the self-attention mechanism of Transformers to capture global relationships. The Graph Convolutional Transformer encoder consists of two main components: graph convolutional layers and self-attention layers. The graph convolutional layers are used to aggregate adjacent nodes’ local features, while the self-attention layers capture the global dependencies between nodes. Through multiple layers of graph convolution and self-attention operations, the encoder can generate high-quality node representations. This combination can better adapt to the characteristics of graph-structured data, improving the model’s performance on graph-related tasks, and enabling our model to effectively harness the graph’s local and global structure, improving its performance on pertinent tasks.

First, we obtain the input feature matrix $F_{s}\in R^{(M+N)\times k}$, with *M* and *N* denoting the miRNA and disease node counts, respectively, and *k* denoting the dimension of the input features.

(8) $$F_{s}=\left[M_{s};D_{s}\right]$$

In the equation, $M_{s}$ and $D_{s}$ denote the miRNA and disease nodes’ similarity matrices, respectively.

In the Graph Convolutional Transformer (GCT), we introduce graph convolutional layers to encode nodes’ local structural patterns by aggregating the neighborhood information, generating more expressive node representations.

(9) $$X_{a}^{\left(0\right)}=F_{s}$$

(10) $$X_{a}^{\left(e\right)}=\sigma \left(\hat{A}X_{a}^{\left(e-1\right)}W^{\left(e\right)}\right)$$

Here, $W^{\left(e\right)}$ represents the trainable weights of the *e*-th graph convolutional layer, while σ denotes the ReLU function.

We introduce the multi-head self-attention layer to model global dependencies between nodes. Nevertheless, in graph convolutional transformers, node representations may converge with increasing encoder depth. The transformer’s inherent self-attention allows nodes to assimilate features from their counterparts, leading to similar feature representations at deep levels [26]. To address this challenge and improve the transformer’s capacity for modeling local relationships, we incorporate a Gaussian bias term into its self-attention layer.

The introduction of the Gaussian bias term is intended to strengthen the transformer’s ability to extract local structural information. By multiplying it with the attention matrix, we encourage nodes to allocate more attention to important nodes that are closer in distance [27,28]. This bias mechanism can help the transformer better capture local patterns and short-range dependencies in the miRNA-disease association graph.

(11) $$Q^{\left(n\right)}=X^{\left(L\right)}W_{Q}^{\left(n\right)},K^{\left(n\right)}=X^{\left(L\right)}W_{K}^{\left(n\right)},V^{\left(n\right)}=X^{\left(L\right)}W_{V}^{\left(n\right)}$$

(12) $$A_{r}^{\left(n\right)}=\mathrm{softmax}(\frac{Q^{\left(n\right)}{\left(K^{\left(n\right)}\right)}^{T}}{\sqrt{d}}+(-|\omega k_{i,j}^{2}+b|))V^{\left(n\right)}$$

(13) $$A_{GT}=\mathrm{Concat}\left(A_{r}^{\left(1\right)},A_{r}^{\left(2\right)},\ldots ,A_{r}^{\left(N\right)}\right)W_{O}$$

In the equations, $X^{\left(L\right)}$ represents the final graph convolutional layer’s output, where $W_{Q}^{\left(n\right)}$, $W_{K}^{\left(n\right)}$, and $W_{V}^{\left(n\right)}$ denote the learnable weight matrices for the *n*-th attention head. The variable *d* represents the dimension of the attention heads, with *N* denoting the attention headcount, $W_{O}$ representing the output weight matrix, $k_{i,j}$ indicating the inter-node distance for nodes *i* and *j*, and ω and *b* serving as learnable scalar parameters. ω controls the scale of the Gaussian bias term, helping the model adapt to different distance metrics, while *b* serves to penalize the weight of a node’s self-attention, preventing nodes from focusing excessively on themselves.

To enhance the model’s stability and generalizability, we introduce residual connections and layer regularization. Residual connections facilitate the smooth flow of gradients, while layer regularization accelerates convergence and mitigates the issue of vanishing gradients.

(14) $$\tilde{A}=\mathrm{LayerNorm}\left(A_{r}+X^{\left(L\right)}\right)$$

(15) $$H_{TR}=\mathrm{LayerNorm}\left(\mathrm{FFN}\left(A_{r}\right)+A_{r}\right)$$

In the equation, FFN represents the feed-forward neural network layer, which consists of two linear transformations and a non-linear activation function.

### 2.5. Fine-Grained Multi-Layer Feature Interaction Gating

To fuse the embeddings generated by the Graph Convolutional Attention Network (GCAN) and Graph Convolutional Transformer encoders, we propose a fine-grained multi-layer feature interaction gating mechanism. This mechanism allows for the gradual integration and refinement of feature representations from the GCAN encoder and Transformer encoder at different levels. We introduce residual connections and feature interaction units tailored to our model. The residual connections add the mean of the input feature-embedding matrices to the fused feature-embedding matrix, promoting gradient flow and feature reuse. The feature interaction units capture the interaction information between different features through non-linear transformations, enhancing the feature representation capability. To mitigate the overfitting problem in deep networks, we apply dropout with a 0.5 probability to regularize the model. The specific representation is as follows:

Specifically, we first compute the gating weight matrix $G^{\left(1\right)}\in R^{N\times D}$ for the first layer, which regulates the fusion ratio of each node across each feature dimension. Then, we use $G^{\left(1\right)}$ to fuse the two feature-embedding matrices $H_{G}$ and $H_{T}$, obtaining the fused feature-embedding matrix $H_{\mathrm{fused}}^{\left(1\right)}\in R^{N\times D}$ for the first layer:

(16) $$G^{\left(1\right)}=\sigma \left(W_{g}^{\left(1\right)}\left[H_{AG};H_{TR}\right]+b_{g}^{\left(1\right)}\right)$$

(17) $$H_{fused}^{\left(1\right)}=G^{\left(1\right)}⊙H_{AG}+\left(1-G^{\left(1\right)}\right)⊙H_{TR}$$

In the equation, $W_{g}^{\left(1\right)}\in R^{2D\times D}$ represents the learnable weight matrix, $b_{g}^{\left(1\right)}\in R^{D}$ denotes the learnable bias vector, with σ denoting the Sigmoid function. The [;] symbol indicates the concatenation operation along the feature dimension, while ⊙ represents the element-wise multiplication operation.

To facilitate gradient flow and feature reuse, we introduce a residual connection that adds the mean of the input feature-embedding matrices to the fused feature-embedding matrix. We also incorporate a feature interaction unit $F^{\left(1\right)}$ to capture the interaction information between different features through non-linear transformations. Furthermore, we apply dropout regularization to the output feature-embedding matrix $H_{out}^{\left(1\right)}$ of the first layer, randomly setting a portion of the elements to zero to reduce overfitting.

(18) $$H_{res}^{\left(1\right)}=H_{fused}^{\left(1\right)}+\left(H_{AG}+H_{TR}\right)/2$$

(19) $$F^{\left(1\right)}=\tanh\left(W_{f}^{\left(1\right)}\left[H_{AG};H_{TR}\right]+b_{f}^{\left(1\right)}\right)$$

(20) $$H_{out}^{\left(1\right)}=H_{res}^{\left(1\right)}+F^{\left(1\right)}$$

(21) $$H_{out}^{\left(1\right)}=\mathrm{Dropout}\left(H_{out}^{\left(1\right)},p\right)$$

In the equation, $W_{f}^{\left(1\right)}$ represents the learnable weight matrix, $b_{f}^{\left(1\right)}$ denotes the learnable bias vector, with tanh being the hyperbolic tangent function.

Finally, we recursively apply the multi-layer gating mechanism, with each layer having its own gating weight matrix, residual connection, and feature interaction unit. This process yields the final fused feature-embedding matrix $H_{out}^{\left(d\right)}$, with *d* denoting the gating mechanism’s total layer count.

(22) $$H_{fused}=H_{out}^{\left(d\right)}$$

The model’s training is optimized using cross-entropy loss, which minimizes the difference between actual and predicted values, thereby enhancing prediction accuracy.

(23) $$P_{f}=-\frac{1}{N}\sum_{v=1}^{N}\left[y_{v}log\left(p_{v}\right)+\left(1-y_{v}\right)log\left(1-p_{v}\right)\right]$$

In the equation, *y* represents the true labels and *p* denotes the model’s predicted labels, with *N* denoting the sample count.

## 3. Results and Discussion

### 3.1. Comparative Analysis with State-Of-The-Art Methods

To comprehensively evaluate the performance of our proposed prediction method, we chose five cutting-edge techniques as baselines: NIMGSA [29], AGAEMD [30], HGANMDA [23], MMGCN [22], and AMHMDA [31]. These cutting-edge approaches serve as benchmarks to assess the effectiveness and efficiency of our model in inferring miRNA-disease links.

- NIMCGCN [29]: employs GCNs to derive features from similarity graphs and integrates a neural inductive matrix completion model to generate a complete miRNA-disease association matrix.
- AGAEMD [30]: considers the attention distribution between nodes in the heterogeneous network and dynamically refines the miRNA functional resemblance profile.
- HGANMDA [23]: leverages attention mechanisms at both node and semantic levels to capture the significance of adjacent nodes and meta-paths, reconstructing the associations between miRNAs and diseases.
- MMGCN [22]: combines GCNs and multi-channel attention mechanisms to extract feature information, adaptively capturing the importance of different features.
- AMHMDA [31]: harnesses GCNs to derive multi-faceted node features from various similarity networks, forming a hypergraph, which is then fused via attention to enable miRNA-disease association inference.

**Figure 2:**
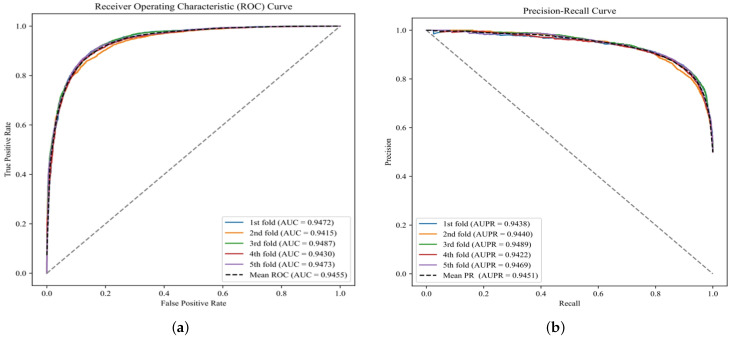
AUC and AUPR curves for five-fold cross-validation. (**a**) AUC pair ratio. (**b**) AUPRC pair ratio.

We employed 5-fold cross-validation on the HMDD v3.2 dataset to rigorously evaluate DGNMDA’s performance. Figure 2 shows that the AUC values for the five-fold cross-validation models are 0.9472, 0.9415, 0.9487, 0.9430, and 0.9473, while the AUPRC values are 0.9438, 0.9440, 0.9489, 0.9422, and 0.9469. These results, along with the data presented in Table 1, highlight the remarkable consistency in our model’s performance.

Through comparative analysis, we have identified two main aspects that limit the performance of existing prediction models. Firstly, current models overlook the higher-order connection patterns and global structural information within the miRNA-disease association network. Secondly, during the feature representation learning process, existing models fail to adequately consider the complex higher-order structure of heterogeneous biological networks. Methods such as NIMGSA, although capable of capturing local node features to a certain extent, are constrained by their neural network encoders and struggle to comprehensively characterize complex biological networks. In contrast, the DGNMDA model demonstrates significant advantages in capturing both global and local information. By employing a graph convolutional Transformer encoder, DGNMDA can simultaneously consider the local structure and global dependencies of nodes, enabling a more comprehensive understanding of the topological properties of miRNA-disease association networks and the discovery of key patterns in higher-order connections. Moreover, DGNMDA incorporates a graph convolutional attention encoder, which adaptively aggregates neighboring node information through an attention mechanism, further enhancing the expressive capability of local features. Furthermore, DGNMDA’s dual heterogeneous graph encoder skillfully captures higher-order structural data within heterogeneous biological networks and precisely localizes complex biological signals at multiple granularity levels, generating more accurate and biologically meaningful feature representations. As a result, the accuracy of miRNA-disease association prediction is significantly improved.

### 3.2. Ablation Study

To comprehensively assess the contributions and importance of various components in the DGNMDA model, we conducted a series of ablation experiments. By designing different model variants and comparing their performance on the HMDD v3.2 dataset, we gained a deep understanding of the roles and efficacy of key components, including the graph convolutional Transformer encoder, graph convolutional attention encoder, and feature interaction gating. We constructed four DGNMDA variants: DGN-A, which removed the self-attention mechanism in the graph convolutional Transformer encoder, retaining only the graph convolutional layers; DGN-B, which replaced the graph convolutional attention encoder with a traditional graph attention encoder; DGN-C, which eliminated the feature interaction gating and directly concatenated the encoder outputs; and DGN-D, which solely employed a conventional Transformer encoder. The experimental results in Table 2 demonstrate that the complete DGNMDA model achieves the best performance across all evaluation metrics. Compared to other variants, DGNMDA exhibits the highest AUC, AUPR, and F1 scores. The ablation experiments reveal the importance and complementarity of different components in the DGNMDA model. Our proposed DGNMDA model can more effectively capture local and global information, while the feature interaction gating adaptively fuses and refines the feature representations from the encoders. These experimental results strongly validate the effectiveness of the DGNMDA model.

### 3.3. Comparison of Single-Source and Multi-Source Features

**Figure 3:**
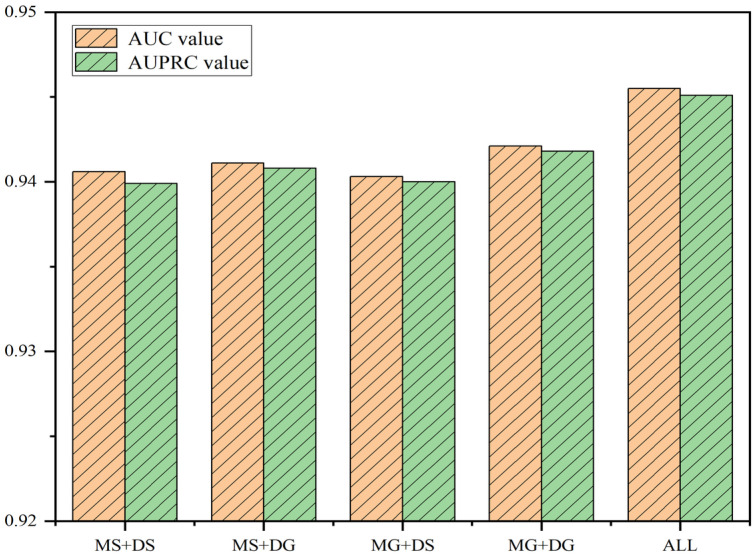
Comparative graph of multi-source and single-source information.

Our model incorporates features from multiple sources as input to our model. Integrating multi-source feature information enhances the precision and stability of miRNA-disease link inference, benefiting from the complementary evidence provided by different data sources. By comprehensively considering miRNA functional similarity, disease semantic similarity, and miRNA-disease association networks, we can more comprehensively characterize the complex biological interactions among miRNAs and diseases and reveal potential regulatory mechanisms. Moreover, integrating multi-source data helps mitigate the sparsity issue encountered when using single data sources and enhances the generalizability of the prediction model. Furthermore, multi-source feature integration can facilitate the discovery of novel association patterns, expanding insights into miRNA functions in diseases and providing important clues for subsequent experimental validation and clinical applications. Therefore, we validated the importance of multi-source information. Table 3 and Figure 3 reveals that the model integrating multi-source information achieves the best performance.

### 3.4. Case Study

**Figure 4:**
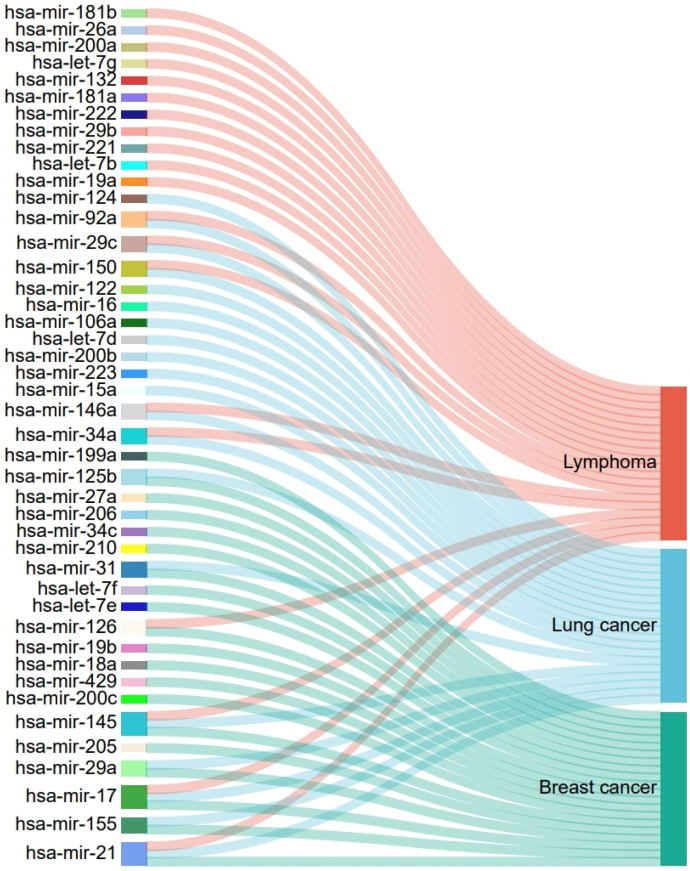
Top 20 miRNAs associated with the three diseases.

Cancer is a multifaceted disorder resulting from the interaction of genetic and environmental elements. miRNAs play crucial roles in cancer progression by regulating oncogene and tumor suppressor gene expression, cell proliferation and apoptosis, invasion, and metastasis [32,33,34,35,36]. Lymphoma is a serious malignant tumor that, if not promptly diagnosed and treated, can adversely affect patients’ health and quality of life in multiple aspects. Research reveals a strong correlation between specific miRNA expression and lymphoma patient outcomes [37,38,39,40]. For instance, elevated miR-21 and miR-155 levels frequently signify an unfavorable outcome, whereas increased miR-34a expression implies an improved prognosis [41]. These miRNAs have the potential to become prognostic biomarkers for guiding risk stratification and treatment decisions in lymphoma patients. Lung cancer, especially NSCLC, is a primary contributor to global cancer mortality [42]. Despite receiving potentially curative treatments, early-stage NSCLC patients still face a recurrence rate of up to 40% within 5 years post-treatment. miRNAs also play significant roles in lung cancer. For example, studies demonstrate that miR-155 and let-7 miRNAs can forecast lung adenocarcinoma outcomes [43,44]. Breast cancer, a prevalent malignancy among women, significantly contributes to female cancer mortality. Research indicates a significant decrease in the levels of specific miRNAs, such as miR-126 and miR-10b, in breast cancer patients [45]. To further validate the capability of the DGNMDA model in predicting miRNA-disease associations, we conducted case studies on three types of cancer: lymphoma, lung cancer, and breast cancer. In the first case study, we focused on lymphoma and lung cancer, using known miRNA-disease associations from HMDD v3.2 as the training set and the associations between these cancers and unknown miRNAs as the test set. We selected the top 20 candidate miRNAs for each cancer based on their prediction scores and verified them using the dbDEMC3.0 database [46]. The results showed that all top 20 predicted miRNAs were confirmed in the database (Table 4, Figure 4), demonstrating DGNMDA’s reliability and outstanding performance in predicting novel cancer-related miRNAs. The second case study aimed to assess DGNMDA’s predictive ability when known association information is lacking, treating breast cancer as a newly emerged disease. We intentionally ignored the known associations between breast cancer and miRNAs during model training, enabling an objective evaluation of DGNMDA’s effectiveness in discovering new miRNA-disease associations. Similarly, we verified the top 20 miRNAs with the highest prediction scores, and the results confirmed their presence in the database (Table 4, Figure 4), further highlighting DGNMDA’s exceptional performance in predicting novel disease-related miRNAs. In summary, through these two case studies, we comprehensively evaluated the performance of the DGNMDA model in predicting cancer-related miRNAs. The experimental results demonstrated that DGNMDA can effectively identify potential disease-related miRNAs for both known cancers and newly emerged diseases. These findings not only validate the reliability and practicality of our model but also provide new insights into understanding the role of miRNAs in cancer development.

### 3.5. Parameter Analysis

Proper hyperparameter tuning enables deep learning models to effectively learn complex miRNA-disease association patterns and excel at inferring novel associations. We performed multiple experiments to identify the ideal hyperparameter settings for maximizing model efficacy and generalizability. Based on the experimental results, the following hyperparameter values were selected: we set the feature-embedding dimension to 512, we set the multi-head attention mechanism to have 4 heads, the number of convolutional layers in the Graph Convolutional Transformer to 2, and the multi-layer gating mechanism set to 2 layers. We employed a dropout rate of 0.5 to mitigate overfitting and enhance DGNMDA’s ability to generalize to unseen data.

#### 3.5.1. Impact of Feature-Embedding Dimension

**Figure 5:**
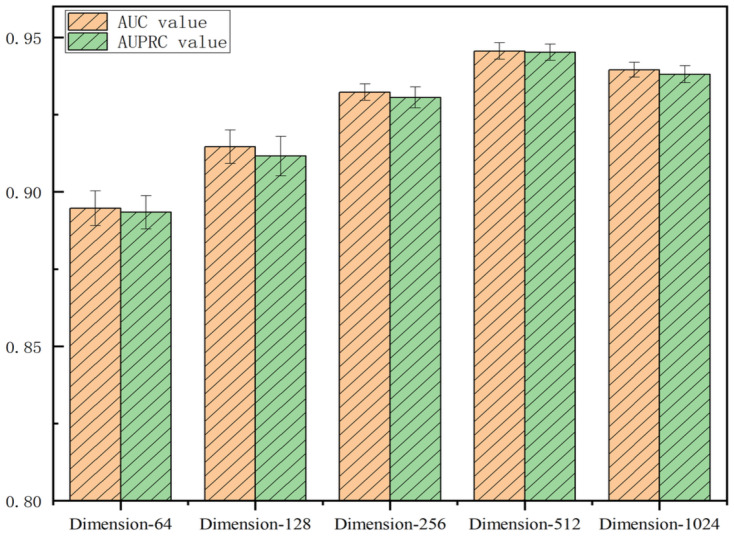
Influence of feature-embedding dimensions on model efficacy.

In deep learning, the choice of feature-embedding dimension significantly influences model performance. The selection of embedding dimension requires a balance between expressive power and computational efficiency. Higher dimensions provide the model with stronger feature-capturing capabilities but also increase computational complexity and memory consumption. Excessively high dimensions may lead to an overly complex model that is difficult to train and generalize. Therefore, in practical applications, it is necessary to balance expressive power and computational efficiency by selecting an appropriate embedding dimension to achieve optimal performance and resource utilization. To investigate the optimal feature-embedding dimension, we designed a series of experiments to evaluate the model’s efficacy across various dimensions. The experimental results, shown in Figure 5, demonstrate that setting the feature-embedding dimension to 512 yields better performance compared to other dimensions.

#### 3.5.2. Experiments on the Number of Multi-Layer Gating Layers

**Figure 6:**
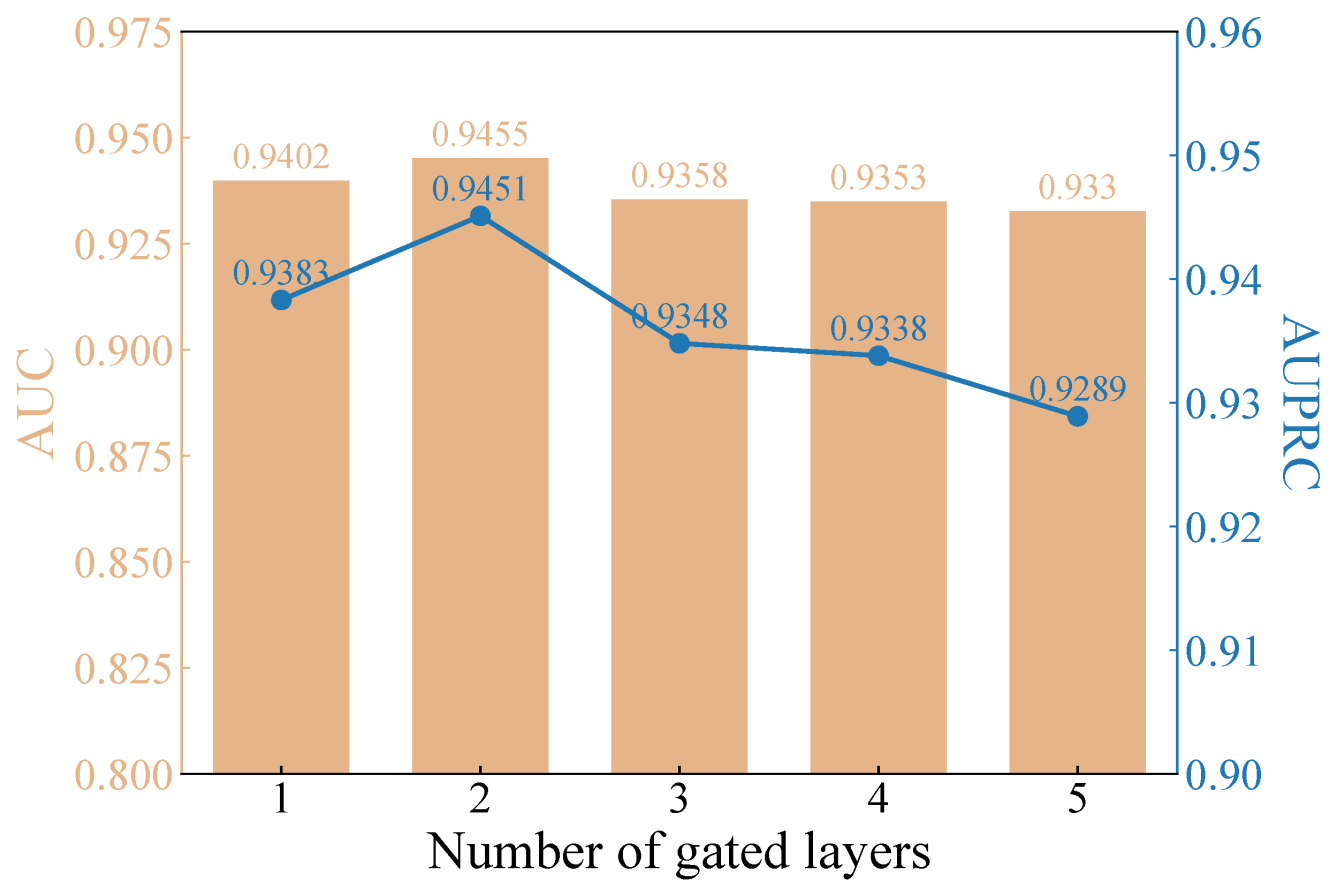
Impact of gating layer count on model performance.

The number of gating layers is a key factor affecting model performance. An insufficient number of gating layers may limit the model’s expressive power, hindering a comprehensive understanding of miRNA-disease associations. On the other hand, too many gating layers, although enabling finer-grained feature fusion, may significantly increase computational complexity and introduce the risk of overfitting. Therefore, selecting the optimal number of gating layers requires balancing the model’s expressive power, computational efficiency, and generalization performance through experimental validation and domain knowledge guidance to find the most suitable configuration for the specific task and dataset, maximizing the potential of the multi-layer gating mechanism. Figure 6 illustrates the experimental findings, indicating that using two layers achieves the best model performance.

#### 3.5.3. Impact of Graph Convolutional Layers and Attention Heads

**Figure 7:**
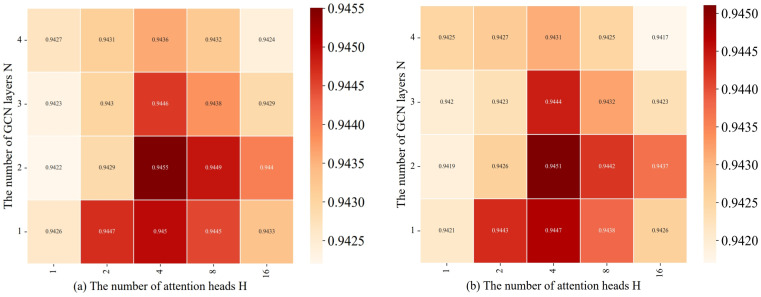
Effect of GCN layer and attention head counts on performance. (**a**) AUC pair ratio. (**b**) AUPRC pair ratio.

Selecting the optimal number of graph convolutional layers and attention heads requires striking a balance between the model’s expressive power, computational efficiency, and generalization performance. Too few graph convolutional layers and attention heads may lead to underfitting and limited expressive power, making it difficult to fully capture the high-order dependencies and diverse interactions between nodes in the graph structure. On the other hand, too many layers and heads may introduce the risk of overfitting and significantly increase computational complexity, affecting the model’s generalization ability and training efficiency. Therefore, it is necessary to find the optimal configuration for the specific task and dataset through experimental validation and domain knowledge guidance. The results of the experiments, presented in Figure 7, demonstrate the impact of different configurations.

## 4. Conclusions

A growing body of experimental evidence reveals significant changes in miRNA expression under various disease conditions, suggesting their pivotal regulatory functions in disease onset and advancement. Motivated by this, our study aims to investigate the prediction of associations between miRNAs and diseases, which is crucial for understanding the molecular mechanisms of diseases and developing new therapeutic strategies. We propose an innovative DGNMDA model that employs a dual heterogeneous graph neural network encoder and incorporates a graph convolutional Transformer and a Graph Convolutional Attention Network (GCAN) encoder. This model can simultaneously capture local structural features and global dependencies, generating more comprehensive node embeddings. Moreover, we design a fine-grained feature interaction gating mechanism to adaptively integrate multi-level features, enhancing the model’s flexibility. To evaluate the model’s performance, we first determine the optimal hyperparameter combination through a series of experiments and then compare DGNMDA with five state-of-the-art models. Our model outperforms the alternatives in terms of AUC and AUPRC metrics. Furthermore, we validate the model’s practicality by assessing its effectiveness in predicting associations for three prevalent and lethal malignancies: lymphoma, lung cancer, and breast cancer. These findings suggest that DGNMDA has the potential to enhance miRNA-disease association prediction precision, deepening our understanding of disease pathogenesis and treatment strategies.

Despite the promising results achieved by DGNMDA, there are still some limitations and challenges. First, the model primarily relies on known association data and similarity measures when constructing similarity networks, and the quality and completeness of this information may affect model performance. In the future, we need to explore more data sources and similarity calculation methods to obtain more comprehensive and reliable similarity information. Second, balancing computational efficiency and prediction performance in real-world applications remains a noteworthy issue. Optimizing model architecture and training strategies to reduce computational overhead while maintaining performance will be a crucial aspect of our further explorations. Additionally, our model currently focuses on binary association predictions between miRNAs and diseases, while the complexity of biological systems extends far beyond this. Incorporating other types of biological entities and constructing more comprehensive multi-entity heterogeneous networks may reveal deeper biological mechanisms. Finally, although our model performs exceptionally well on benchmark datasets, more validation and refinement are needed for practical applications. Comparing model predictions with wet-lab experimental results and iteratively optimizing the model based on expert knowledge will help improve the model’s interpretability and credibility. Simultaneously, developing user-friendly visualization tools to facilitate the use and interpretation of model results by biologists and medical researchers is a crucial step in promoting the model’s application in real-world scenarios.

## Figures and Tables

**Table 1:** Comparative evaluation of alternative approaches.

Methods	ACC	F1-Score	Recall	Precision	AUC	AUPRC
NIMCGCN	0.8131	0.8148	0.8220	0.8076	0.8945	0.8926
AGAEMD	0.8502	0.8507	0.8544	0.8481	0.9270	0.9286
HGANMDA	0.8489	0.8481	0.8433	0.8529	0.9265	0.9253
MAGCN	0.8483	0.8473	0.8425	0.8533	0.9245	0.9268
AMHMDA	0.8648	0.8623	0.8539	0.8755	0.9411	0.9403
**DGNMDA**	**0.8773**	**0.8800**	**0.8768**	**0.8896**	**0.9455**	**0.9451**

Bold values indicate the best performance.

**Table 2:** Comparative evaluation of alternative approaches.

Methods	DGN-A	DGN-B	DGN-C	DGN-D	DGNMDA
AUC	0.9367	0.9398	0.9392	0.9382	**0.9455**
AUPR	0.9358	0.9374	0.9383	0.9367	**0.9451**

Bold values indicate the best performance.

**Table 3:** Multi-source feature experiment.

Metrics	MS+DS	MS+DG	MG+DS	MG+DG	ALL
AUC	0.9406	0.9411	0.9403	0.9421	**0.9455**
AUPR	0.9399	0.9408	0.9400	0.9418	**0.9451**

Bold values indicate the best performance.

**Table 4:** Predicted top 20 miRNAs: highest associations with lymphoma, lung cancer, and breast cancer.

Cancer: Lymphoma					
Rank	miRNA	Evidence	Rank	miRNA	Evidence
1	hsa-mir-21	dbDEMC	11	hsa-mir-150	dbDEMC
2	hsa-mir-34a	dbDEMC	12	hsa-mir-29b	dbDEMC
3	hsa-mir-17	dbDEMC	13	hsa-mir-222	dbDEMC
4	hsa-mir-92a	dbDEMC	14	hsa-mir-181a	dbDEMC
5	hsa-mir-145	dbDEMC	15	hsa-mir-29c	dbDEMC
6	hsa-mir-19a	dbDEMC	16	hsa-mir-132	dbDEMC
7	hsa-mir-126	dbDEMC	17	hsa-let-7g	dbDEMC
8	hsa-mir-146a	dbDEMC	18	hsa-mir-200a	dbDEMC
9	hsa-let-7b	dbDEMC	19	hsa-mir-26a	dbDEMC
10	hsa-mir-221	dbDEMC	20	hsa-mir-181b	dbDEMC
**Cancer: Lung cancer**					
**Rank**	**miRNA**	**Evidence**	**Rank**	**miRNA**	**Evidence**
1	hsa-mir-21	dbDEMC	11	hsa-mir-145	dbDEMC
2	hsa-mir-155	dbDEMC	12	hsa-mir-125b	dbDEMC
3	hsa-mir-17	dbDEMC	13	hsa-mir-16	dbDEMC
4	hsa-mir-34a	dbDEMC	14	hsa-mir-29a	dbDEMC
5	hsa-mir-146a	dbDEMC	15	hsa-mir-31	dbDEMC
6	hsa-mir-15a	dbDEMC	16	hsa-mir-122	dbDEMC
7	hsa-mir-223	dbDEMC	17	hsa-mir-150	dbDEMC
8	hsa-mir-200b	dbDEMC	18	hsa-mir-29c	dbDEMC
9	hsa-let-7d	dbDEMC	19	hsa-mir-92a	dbDEMC
10	hsa-mir-106a	dbDEMC	20	hsa-mir-124	dbDEMC
**Cancer: Breast cancer**					
**Rank**	**miRNA**	**Evidence**	**Rank**	**miRNA**	**Evidence**
1	hsa-mir-21	dbDEMC	11	hsa-mir-126	dbDEMC
2	hsa-mir-155	dbDEMC	12	hsa-let-7e	dbDEMC
3	hsa-mir-17	dbDEMC	13	hsa-let-7f	dbDEMC
4	hsa-mir-29a	dbDEMC	14	hsa-mir-31	dbDEMC
5	hsa-mir-205	dbDEMC	15	hsa-mir-210	dbDEMC
6	hsa-mir-145	dbDEMC	16	hsa-mir-34c	dbDEMC
7	hsa-mir-200c	dbDEMC	17	hsa-mir-206	dbDEMC
8	hsa-mir-429	dbDEMC	18	hsa-mir-27a	dbDEMC
9	hsa-mir-18a	dbDEMC	19	hsa-mir-125b	dbDEMC
10	hsa-mir-19b	dbDEMC	20	hsa-mir-199a	dbDEMC

## Data Availability

The miRNA-disease association data used in this study were obtained from the publicly available HMDD v3.2 database, accessed on 28 February 2024, through the website http://www.cuilab.cn/hmdd.
